# Correlation of a Decline in Aerobic Capacity with Development of Emphysema in Patients with Chronic Obstructive Pulmonary Disease: A Prospective Observational Study

**DOI:** 10.1371/journal.pone.0125053

**Published:** 2015-04-24

**Authors:** Wakako Yamasawa, Sadatomo Tasaka, Tomoko Betsuyaku, Kazuhiro Yamaguchi

**Affiliations:** 1 Division of Pulmonary Medicine, Keio University School of Medicine, Tokyo, Japan; 2 Department of Laboratory Medicine, Keio University School of Medicine, Tokyo, Japan; 3 Comprehensive and Internal Medicine, Tokyo Women's Medical University Medical Center East, Tokyo, Japan; University Hospital Freiburg, GERMANY

## Abstract

In patients with COPD, CT assessment of emphysema and airway disease is known to be associated with lung function and 6-minute walk distance. However, it remains to be determined whether low attenuation area (LAA) on CT is associated with aerobic capacity assessed using cardiopulmonary exercise testing (CPET). In this prospective observational study, we repeatedly conducted high-resolution CT and CPET using a treadmill in 81 COPD patients over a median interval of 3.5 years. Two investigators independently scored LAA on images obtained at the aortic arch level, tracheal bifurcation level, and supradiaphragmatic level. Grades for the images of each lung were added to yield the total LAA score. Total LAA score was negatively correlated with peak aerobic capacity ($\dot{\text{V}}{\text{O}}_{2}$) (p<0.001, r = -0.485). LAA scores of the upper (aortic arch level) and the lower (supradiaphragmatic level) lungs were both significantly associated with peak $\dot{\text{V}}{\text{O}}_{2}$. There was a significant correlation between total LAA score and peak CO_2_ output ($\dot{\text{V}}{\text{CO}}_{2}$) (p<0.001, r = -0.433). Total LAA score was correlated with oxygen saturation at peak exercise (p<0.001, r = -0.634) and the estimated dead space fraction (p<0.001, r = 0.416). The mean annual change in total LAA score was significantly correlated with those in peak $\dot{\text{V}}{\text{O}}_{2}$ (p<0.001, r = -0.546) and peak $\dot{\text{V}}{\text{CO}}_{2}$ (p<0.001, r = -0.488). The extent of emphysema measured by CT was associated with the results of CPET. The time-dependent changes in CPET data were also correlated with that in total LAA score. CT assessment could be a non-invasive tool to predict aerobic capacity in patients with COPD.

## Introduction

Impaired exercise capacity is frequently observed in patients with chronic obstructive pulmonary disease (COPD) [1] and is traditionally associated with impaired lung function [2–4]. There is, however, increasing recognition that spirometric measures of lung function alone do not explain all the variance found in clinical measures of disease. A growing body of evidence has revealed that computed tomography (CT) assessment of emphysema and airway disease is helpful for understanding the disease pathophysiology, and the findings are associated with clinical parameters such as exercise capacity [5–7]. It was also reported that CT measures of emphysema and airway disease relate to COPD exacerbation frequency [8]. In addition, emphysema measured by CT significantly influenced BODE, an index of clinical outcome of patients with COPD [9]. Whereas CT is increasingly performed to quantitatively assess emphysema as well as airway disease in COPD [5, 10–13], studies of their contribution to exercise capacity are still limited [7, 14]. There have been few reports about the association between CT measures of emphysema and exercise capacity assessed by walking tests [7, 14].

The 6-minute walk test (6MWT) is commonly used to measure the exercise capacity in patients with COPD. Although the 6MWT results are known to be a good predictor of mortality in patients with severe COPD [15], 6MWT is a less quantitative approach, because the results are often influenced by the effort and willingness of the subjects. There is a significant but modest correlation between oxygen uptake ($\dot{\text{V}}$ o_2_) at peak exercise (peak $\dot{\text{V}}$ o_2_) and walk distance [16]. In addition, the extent of emphysema measured by CT imaging is associated with the walking distance in patients with COPD [17]. However, no study has yet examined the predictive value of the extent of emphysema on CT images for the exercise capacity in COPD patients.

In this study, we evaluated the relation of emphysema to exercise capacity assessed by cardiopulmonary exercise testing (CPET) using a treadmill. Instead of the 6MWT, we applied the treadmill test, which was expected to allow the estimation of exercise capacity in a more quantitative manner, in this study. Based on prior studies, we hypothesized that high-resolution CT (HRCT) measures of emphysema predict exercise capacity assessed by CPET independent of lung function. We first examined whether the extent of emphysema on HRCT is associated with exercise capacity and lung function. In addition, we evaluated the association between temporal changes in emphysema on HRCT images and those in exercise capacity and lung function.

## Methods

### Subjects enrolled

Participants were selected from the subjects who had been referred to the outpatient clinic of our Keio University hospital on suspicion of a variety of smoking-related pulmonary diseases. Among them, the subjects, who had an airflow limitation defined as the ratio of forced expiratory volume in 1 s to forced expiratory vital capacity [FEV_1_/FVC] <0.7 and a significant lifelong history of smoking exceeding 10 pack-years, were evaluated for inclusion in this prospective observational study. They underwent HRCT, CPET, and lung function tests on the occasion of study entry, and these examinations were repeated at an interval of at least 2 years. Patients continued all medications and were started on new ones if necessary. All participants provided written informed consent. The study protocol was approved by the ethical committee of Keio University School of Medicine. Demographical data, smoking and medical history were collected through interview.

### Pulmonary function tests

FEV_1_ and FEV_1_/FVC were measured according to the American Thoracic Society recommendations (Chestac-9800, Chest M.I., Tokyo, Japan) [18]. The results were expressed as absolute and percent predicted values. Lung volumes were obtained using body plethysmography.

### Cardiopulmonary exercise testing

The treadmill test was performed in incorporation with increases in speed and incline for producing an incremental augmentation of the work rate. The treadmill is started at a speed of 2.74 km/hr and at an incline of 10% [19]. At three minute intervals, the incline of the treadmill was increased by 2%. Heart rate was monitored with a 12-lead ECG, and blood pressure was measured by the cuff technique (STS-8000, Nihon Kohden, Tokyo, Japan). Minute ventilation ($\dot{\text{V}}$ e) and its components were measured using a pneumotachograph (Vmax 229, Nihon Kohden, Tokyo, Japan). The concentrations of expired oxygen and carbon dioxide were analyzed breath by breath. These measures and flow signals were integrated to yield 30-s averages of $\dot{\text{V}}$ e, tidal volume, respiratory rate, $\dot{\text{V}}$ o_2_, and carbon dioxide output ($\dot{\text{V}}$ co_2_). The predicted maximal $\dot{\text{V}}$ o_2_ was calculated using standard equations [20]. Maximal voluntary ventilation was directly determined for > 12 s. Heart rate reserve was calculated as the predicted maximal heart rate (maximal heart rate = 220 beats/min—age) minus the observed peak heart rate. Dead-space fraction ($\dot{\text{V}}$ d/$\dot{\text{V}}$ t) was estimated based on end-tidal CO_2_ measurements at peak exercise.

### High-resolution computed tomography

All HRCT scans were conducted with the subject supine (HiSpeed NX/i; GE Yokogawa Medical Systems, Tokyo, Japan) using 1.0-mm-thick sections taken at 1-cm intervals throughout the entire thorax. Two experienced pulmonologists (W.Y. and S.T.) independently evaluated all HRCT images with blindness to any information regarding the pulmonary function tests and cardiopulmonary exercise testing.

The presence of emphysema on HRCT was defined as well demarcated areas of decreased attenuation, as compared with contiguous normal lung tissue, with margin by a very thin (<1 mm) or no wall and/or multiple bullae (>1 cm) with upper zone predominance. In patients with emphysema, low attenuation areas (LAA) were visually assessed by the method of Goddard and colleagues [21]. Briefly, the whole lung was divided into six zones (left and right zones in the upper, middle and lower lung fields). Two investigators independently scored LAA on the images obtained at the aortic arch level, tracheal bifurcation level, and supradiaphragmatic level to evaluate emphysema in the upper, middle and lower lung fields, respectively. LAA in each image section was scored on a scale of 0 to 4, where 0 = no LAA, 1 = 1–25%, 2 = 26–50%, 3 = 51–75%, and 4 = 76–100% LAA. Grades for the images of each zone were added to yield the total LAA score.

### Statistical Analysis

For continuous variables, the data are presented as the mean ± SE. Pearson’s correlation coefficients were used to assess relationships between the parameters. For multiple comparisons, analysis of variance was performed with the *post hoc* Scheffe’s test to determine significant differences. A probability value less than 0.05 was considered to be significant. Mean annual changes of the parameters were calculated by dividing the difference of the value by the interval of the measurements.

## Results

### Patient characteristics and baseline data

The clinical characteristics of the 81 subjects studied are shown in Table 1. Eighty were male and the age was 69 ± 2 years. The body mass index (BMI) was 22.8 ± 0.3 kg/m^2^, which was similar to the average among the elderlies in Japan. The study subjects contained 34 ex-smokers and 47 current-smokers at study entry. Seventeen patients quitted smoking during the observation period. Nineteen of the study subjects received no medical treatment and 62 were taking an inhaled anticholinergic or β_2_-agonist, or both. The median interval of evaluation was 3.5 years, ranging from 2.0 to 5.1 years. During the observation, eight patients experienced an exacerbation, defined as sustained worsening of symptoms in comparison with their usual stable state. None of the study subjects suffered two or more exacerbations. The mean annual decline in FEV_1_ was 0.032 L, while its standard deviation was 0.106 L. There were 8 patients with an annual decline exceeding the mean plus one standard deviation. These patients were assumed to be the rapid decliners concerning FEV_1_.

**Table 1:** Baseline demographic and physiological data.

Age (yr)	69 ± 2
Male/female	80/1
Body mass index (kg/m^2^)	22.8 ± 0.3
Smoking (pack-year)	58 ± 6
Current smoker	47
Ex-smoker	34
Severity of COPD	
GOLD grade I	16
II	39
III	21
IV	5
Medications	
None	19
Anticholinergic	34
β_2_-agonist	25
ICS/LABA combination	3
Lung function tests	
VC (L)	3.62 ± 0.09
%VC	98.0 ± 2.0
FEV_1_ (L)	1.83 ± 0.08
FEV_1_/FVC (%)	51.6 ± 1.4
%FEV_1_ (% pred.)	63.5 ± 2.5
RV/TLC (%)	38.6 ± 1.1
DLco (mL/min/Torr)	17.4 ± 0.7
%DLco (%pred.)	63.8 ± 2.7
DLco/Va (mL/min/Torr/L)	3.66 ± 0.17
%DLco/Va (%pred.)	62.9 ± 2.8

Data are presented as mean ± SE

CPET data obtained at the study entry are summarized in Table 2. In comparison with the predicted values, $\dot{\text{V}}$ o_2_ was impaired. Although oxygen saturation was preserved, many patients complained of severe dyspnea at the end of the test. Eighteen patients stopped CPET due to leg fatigue or other muscular problems.

**Table 2:** Cardiopulmonary exercise testing data and LAA scores at study entry.

Cardiopulmonary exercise testing	
Peak $\dot{\text{V}}$ e (L/min)	57.5 ± 1.7
Peak $\dot{\text{V}}$ t (L)	1.65 ± 0.05
Peak respiratory rate (BPM)	32.9 ± 0.7
$\dot{\text{V}}$ d/$\dot{\text{V}}$ t (peak)	0.185 ± 0.007
$\dot{\text{V}}$ e/MVV (peak)	0.88 ± 0.04
Peak $\dot{\text{V}}$ o_2_ (L/min)	1.37 ± 0.04
Peak $\dot{\text{V}}$ o_2_/kg (mL/kg/min)	21.9 ± 0.6
$\dot{\text{V}}$ o_2_ (%pred.)	80.0 ± 3.0
$\dot{\text{V}}$ o_2_/kg (%pred.)	85.1 ± 3.0
Peak $\dot{\text{V}}$ co_2_ (L/min)	1.52 ± 0.05
Peak heart rate (BPM)	132 ± 2
SpO_2_ at peak exercise	89.8 ± 0.6
Borg scale at the end of the test	8.3 ± 0.3
LAA scores	
Total score	10.50 ± 0.89
Upper lung fields	4.05 ± 0.35
Middle lung fields	3.51 ± 0.30
Lower lung fields	2.95 ± 0.29

Data are presented as mean ± SE

LAA scores obtained at study entry are also summarized in Table 2. LAA score in the upper lung field was significantly greater than that in the lower lung field (p<0.05).

### Association between cardiopulmonary exercise testing data and LAA scores

**Fig 1:**
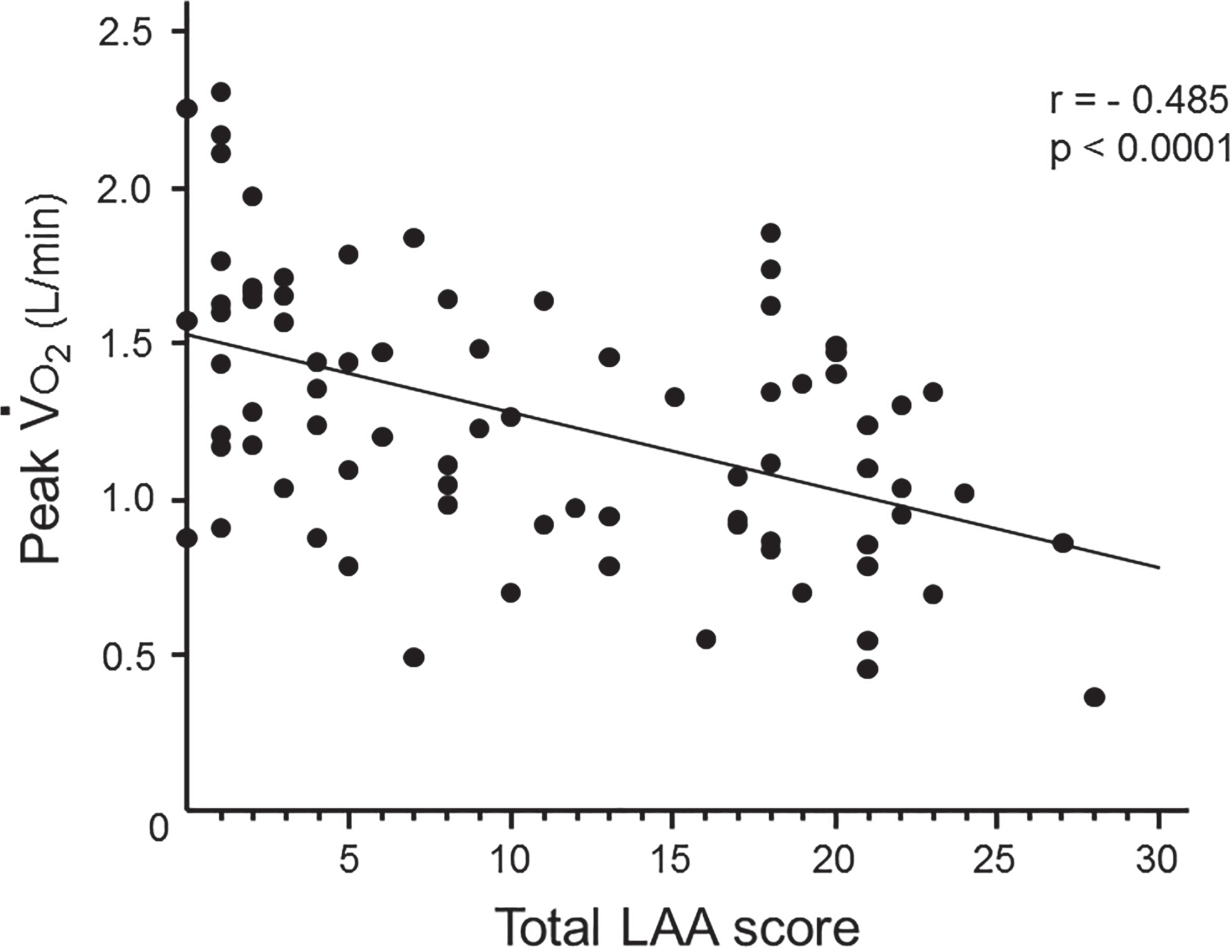
Association between aerobic capacity and emphysematous changes. The aerobic capacity at peak exercise (peak $\dot{\text{V}}$ o_2_) was inversely correlated with total LAA scores (p<0.0001, r = -0.485).

We evaluated the association between CPET parameters and LAA scores on HRCT obtained at the study entry. As shown in Fig 1, oxygen uptake at peak exercise (peak $\dot{\text{V}}$ o_2_) was inversely correlated with total LAA score (p<0.0001, r = -0.485). LAA scores of the upper (aortic arch level) and lower (supradiaphragmatic level) lungs were both significantly associated with peak $\dot{\text{V}}$ o_2_ (data not shown).

**Fig 2:**
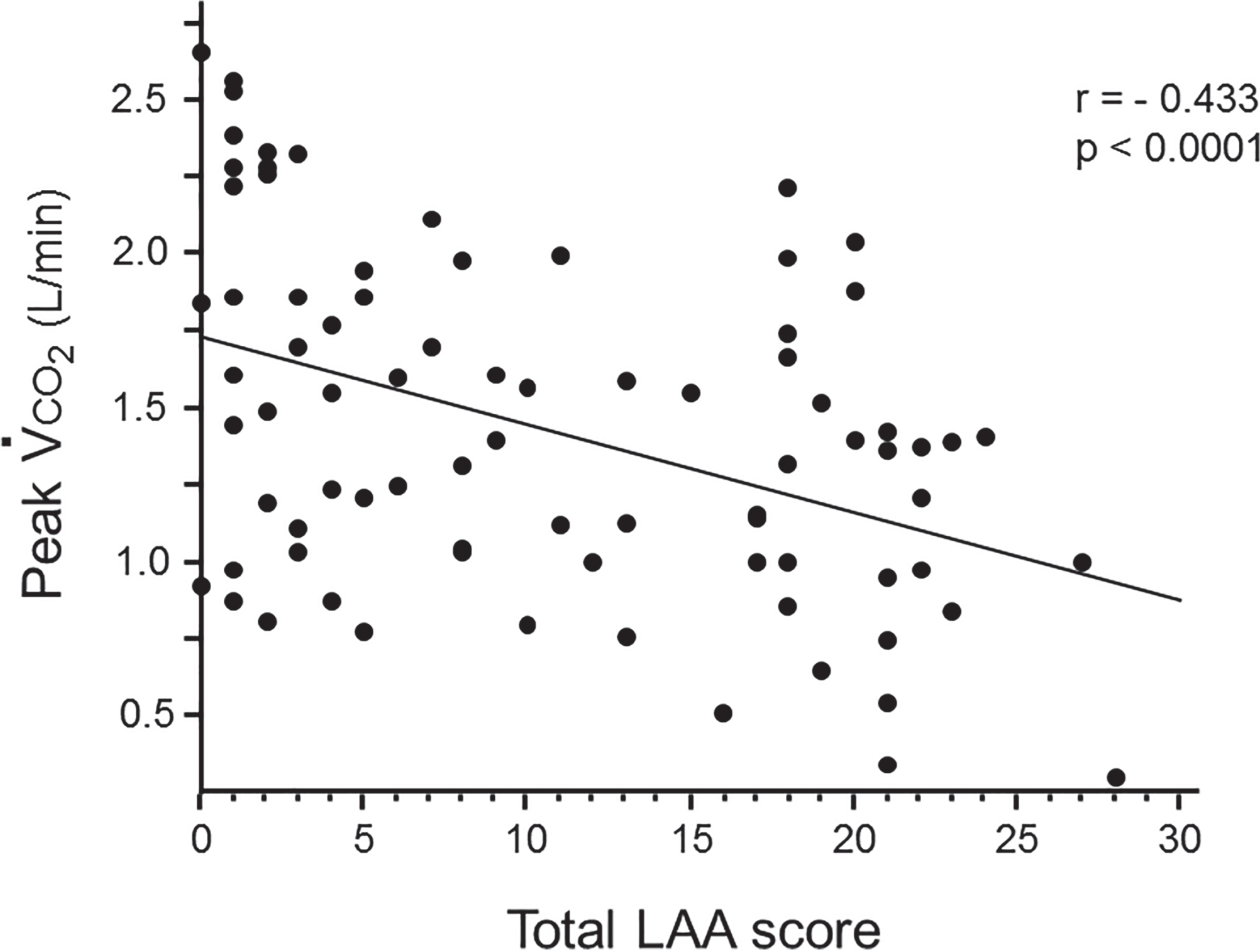
Relationship between CO_2_ production and emphysematous changes. The CO_2_ production at the peak exercise (peak $\dot{\text{V}}$ co_2_) was inversely correlated with the total LAA scores (p<0.0001, r = -0.433).

The association between total LAA score and CO_2_ production ($\dot{\text{V}}$ co_2_) at peak exercise (peak $\dot{\text{V}}$ co_2_) is shown in Fig 2. Peak $\dot{\text{V}}$ co_2_ was negatively correlated with total LAA score (p<0.0001, r = -0.433), which indicated that the development of emphysematous change might result in alveolar hypoventilation, leading to decreased CO_2_ excretion.

**Fig 3:**
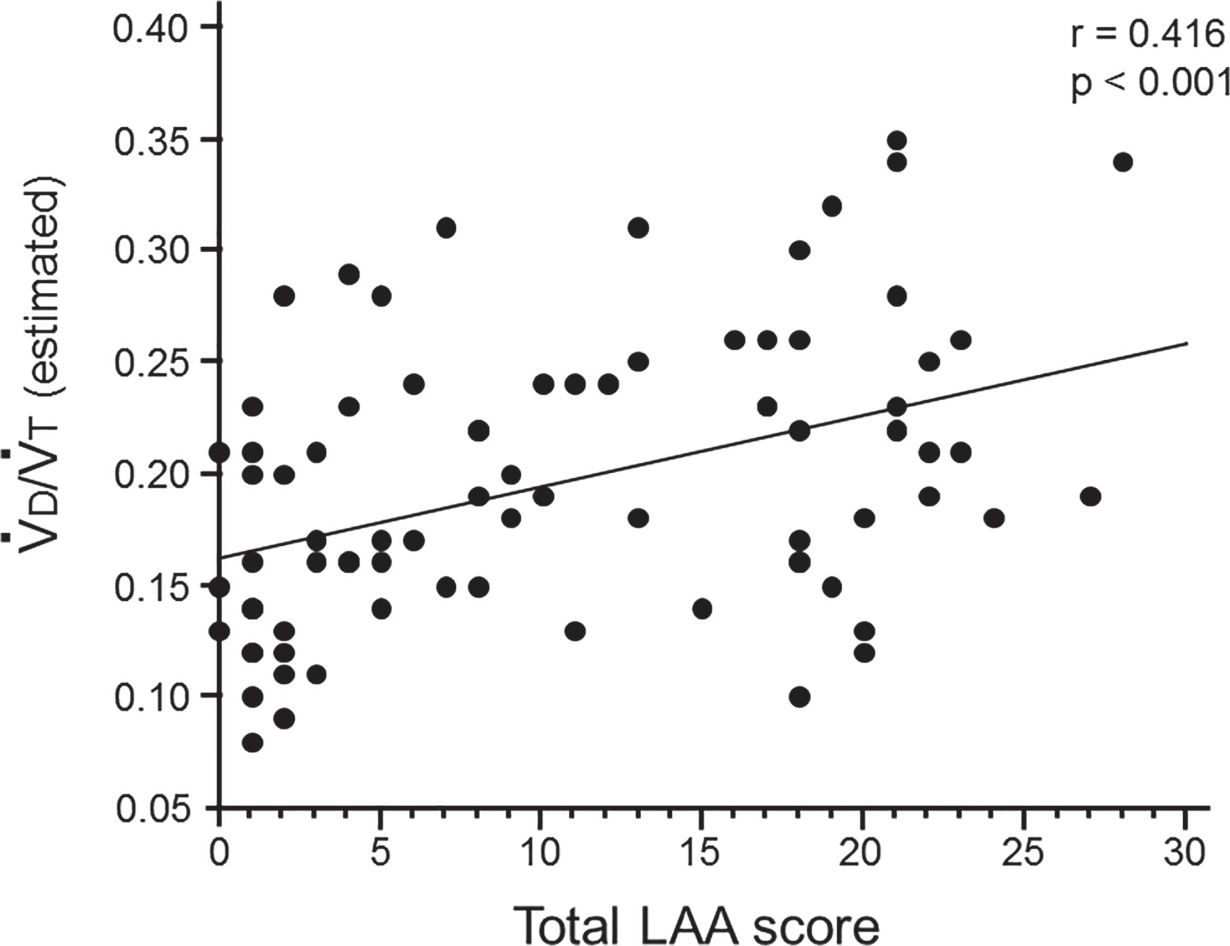
Association between the dead space and emphysema. The total LAA scores were positively correlated with the estimated dead space fractions ($\dot{\text{V}}$ d/$\dot{\text{V}}$ t) (p<0.001, r = 0.416).

Total LAA score was positively correlated with estimated $\dot{\text{V}}$ d/$\dot{\text{V}}$ t (p<0.001, r = 0.416) (Fig 3). The extent of emphysema could be associated with increased dead space. There was no significant correlation between $\dot{\text{V}}$ e at peak exercise and total LAA score (data not shown).

**Fig 4:**
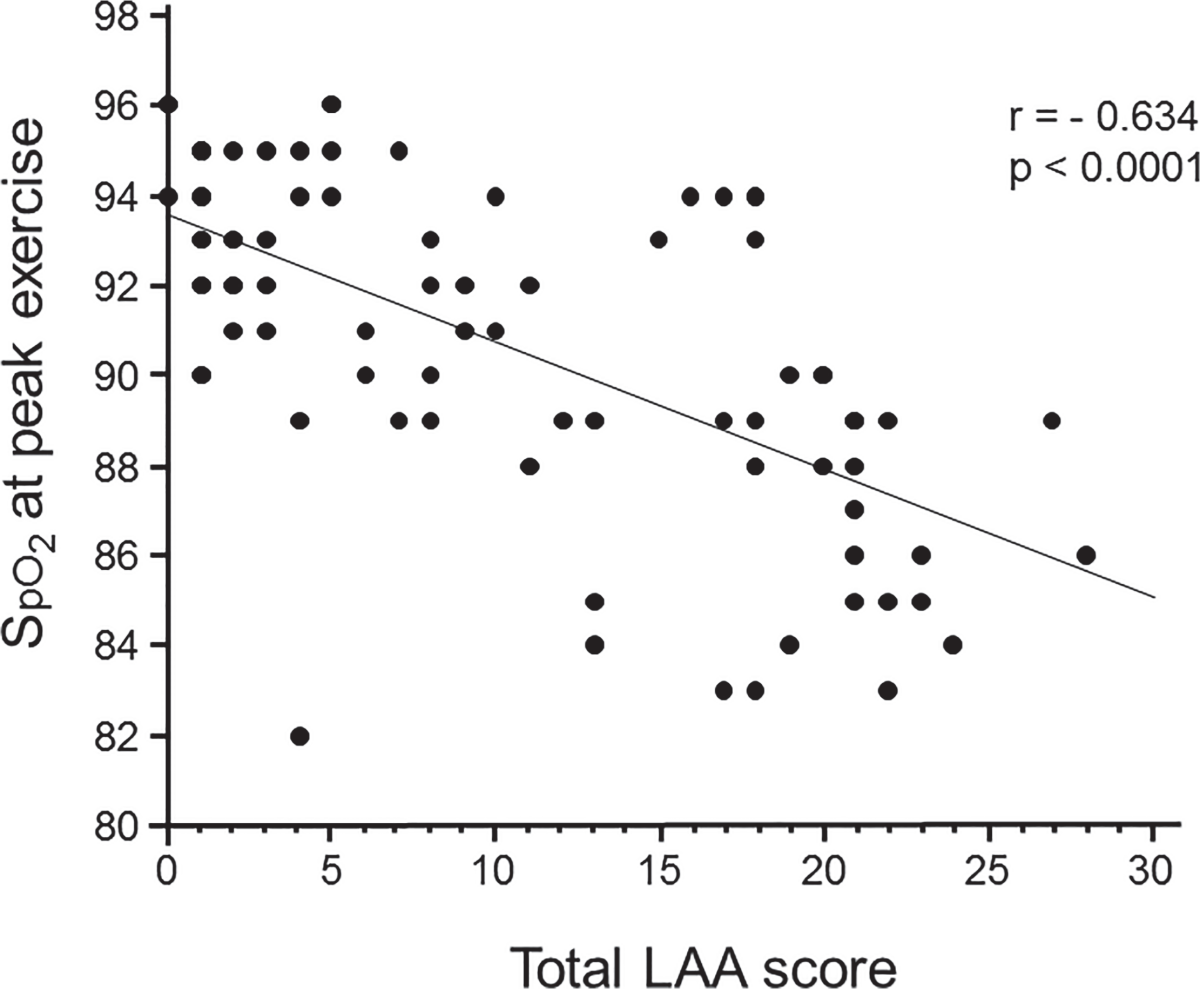
Association between emphysematous changes and oxygen desaturation during exercise. The oxygen saturation (SpO_2_) at the peak exercise was inversely correlated with total LAA scores (p<0.0001, r = -0.634).

As shown in Fig 4, SpO_2_ at peak exercise was inversely correlated with total LAA score (p<0.0001, r = -0.634), which indicated that the development of emphysematous change might be associated with oxygen desaturation during exercise.

### Correlations between cardiopulmonary exercise testing data and other clinical parameters

We evaluated the association between CPET parameters and other clinical parameters, such as age, BMI, FEV_1_ as a percentage of predicted (%FEV_1_), and diffusing capacity as a percentage of predicted (%DLco/Va), obtained at the study entry. The correlation coefficients are summarized in Table 3. In addition to total LAA score, both peak $\dot{\text{V}}$ o_2_ and peak $\dot{\text{V}}$ co_2_ were correlated positively with BMI, %FEV_1_, %DLco/Va and negatively with age. Estimated $\dot{\text{V}}$ d/$\dot{\text{V}}$ t at peak exercise was correlated with age, BMI, and %FEV_1_, but not with diffusing capacity. SpO_2_ at peak exercise was correlated with %FEV_1_ and %DLco/Va, although it was not associated with age or BMI of the patients. It was indicated that these factors might influence the results of CPET as the confounders.

**Table 3:** Correlation coefficients between cardiopulmonary exercise testing data and other clinical parameters at study entry.

	Peak $\dot{\text{V}}$ o_2_	Peak $\dot{\text{V}}$ co_2_	Estimated $\dot{\text{V}}$ d/$\dot{\text{V}}$ t at peak exercise	SpO_2_ at peak exercise
Age	r = -0.483 p<0.0001	r = -0.466 p<0.0001	r = 0.377 p<0.001	r = -0.070 p = 0.534
Body mass index	r = 0.411 p<0.0001	r = 0.391 p<0.001	r = -0.372 p<0.001	r = 0.073 p = 0.516
Total LAA score	r = -0.485 p<0.0001	r = -0.433 p<0.0001	r = 0.416 p<0.001	r = -0.634 p<0.0001
%FEV_1_ (% pred.)	r = 0.419 p<0.0001	r = 0.443 p<0.0001	r = -0.571 p<0.0001	r = 0.321 p<0.01
%DLco/Va (%pred.)	r = 0.323 p<0.01	r = 0.256 p<0.05	r = -0.205 p = 0.066	r = 0.446 p<0.0001

Because %FEV_1_ was correlated with peak $\dot{\text{V}}$ o_2_ and peak $\dot{\text{V}}$ co_2_, it was indicated that the airflow limitation in COPD could be associated with exercise capacity. Peak $\dot{\text{V}}$ o_2_ in patients with GOLD grade I, II, III, and IV were 1.53 ± 0.07, 1.39 ± 0.07, 1.12 ± 0.06, and 0.91 ± 0.15 L/min, respectively. Peak $\dot{\text{V}}$ o_2_ was significantly higher in patients with GOLD grade I than in those with grade III and IV (p<0.05). Peak $\dot{\text{V}}$ co_2_ in patients with GOLD grade I, II, III, and IV were 1.72 ± 0.09, 1.57 ± 0.09, 1.19 ± 0.07, and 0.85 ± 0.15 L/min, respectively. There were significant differences in peak $\dot{\text{V}}$ co_2_ between GOLD grade I and grades III and IV and between GOLD grade II and IV (p<0.05).

Based on multivariate analyses, we calculated the partial correlation coefficient (r_p_) to exclude the effects of age, BMI, airflow limitation (%FEV_1_), and diffusing capacity (%DLco/Va). After excluding the effects of these factors, total LAA score still had significant correlations with peak $\dot{\text{V}}$ o_2_ (r_p_ = -0.363), peak $\dot{\text{V}}$ co_2_ (r_p_ = -0.289), and SpO_2_ at peak exercise (r_p_ = -0.348), but not with estimated $\dot{\text{V}}$ d/$\dot{\text{V}}$ t at peak exercise (r_p_ = 0.189).

### Association between longitudinal changes in CPET data and that in LAA score

**Fig 5:**
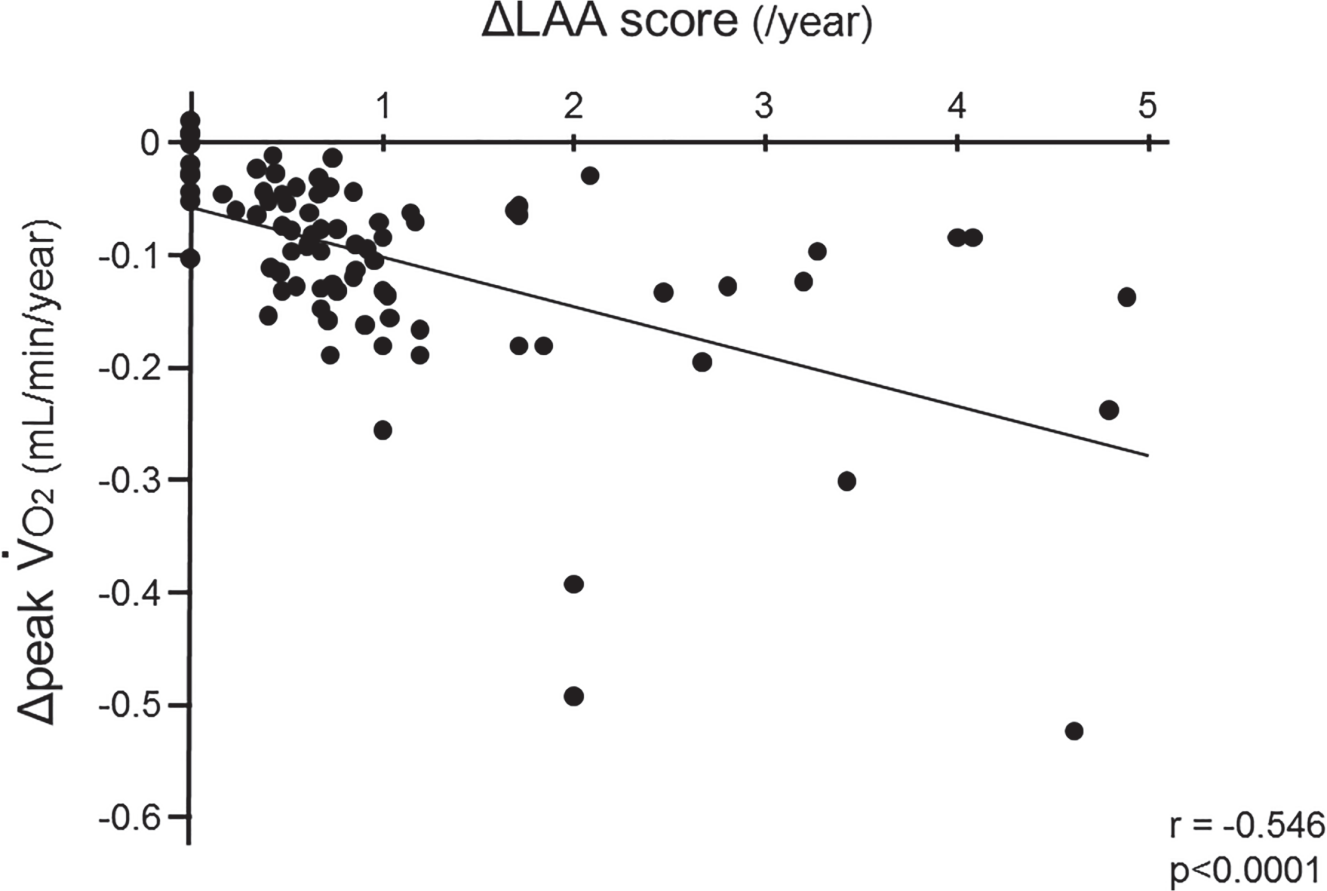
Relationship between the decline in aerobic capacity and progression of emphysema. The mean annual changes in peak $\dot{\text{V}}$ o_2_ (Δp$\dot{\text{V}}$ o_2_) were significantly correlated with those in the total LAA scores (ΔLAA) (p<0.0001, r = -0.546).

We examined whether the longitudinal changes in CPET data were associated with those in total LAA score. As shown in Fig 5, the mean annual change in peak $\dot{\text{V}}$ o_2_ (Δp$\dot{\text{V}}$ o_2_) was significantly correlated with that in total LAA score (ΔLAA) (p<0.0001, r = -0.546), which indicated that aerobic capacity could decline in proportion to the development of emphysematous changes.

**Fig 6:**
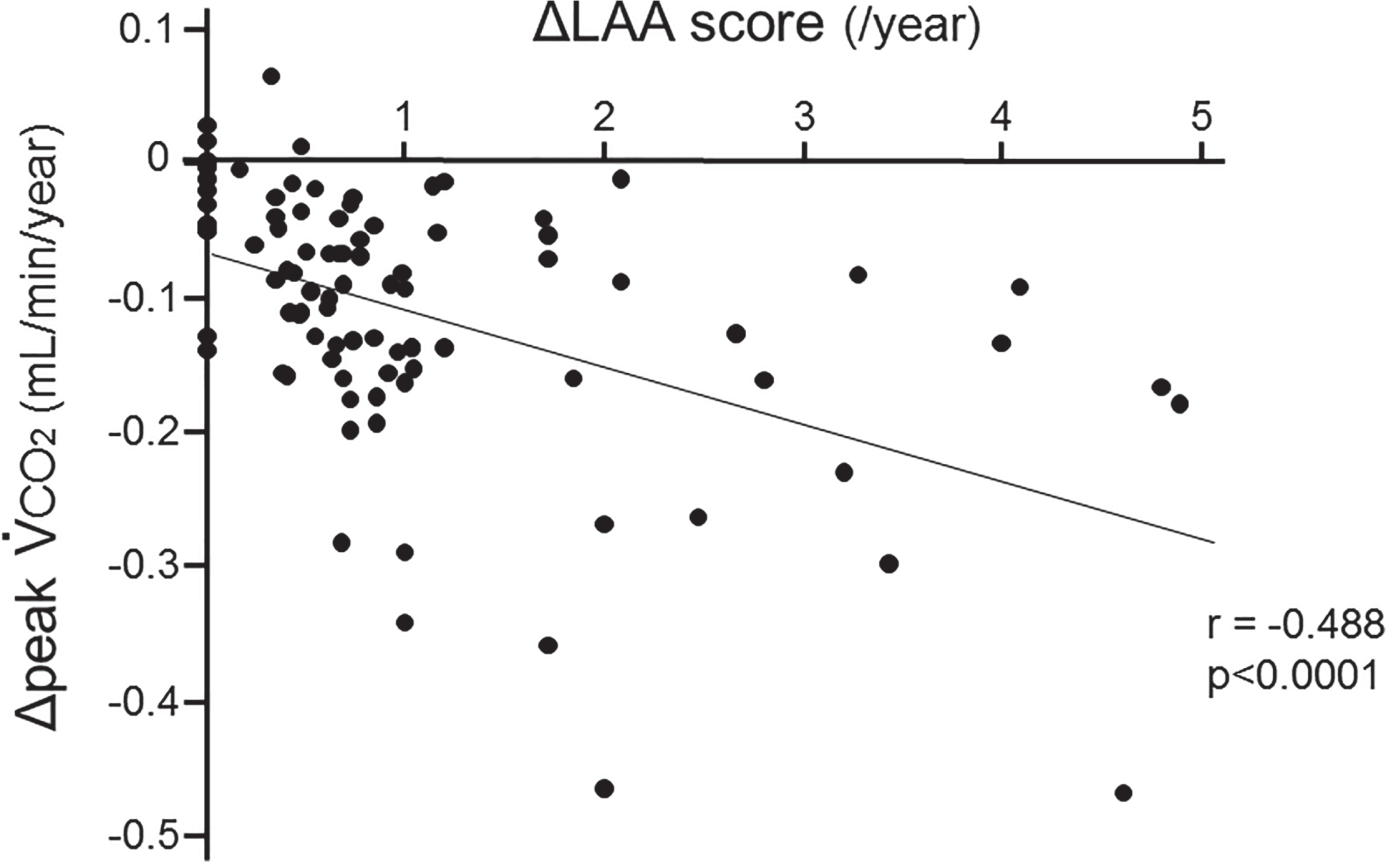
Association between the decline in CO2 production and development of emphysema. There was a significant correlation between the mean annual changes in peak $\dot{\text{V}}$ co_2_ (Δp$\dot{\text{V}}$ co_2_) and those in the total LAA scores (ΔLAA) (p<0.0001, r = -0.488).

The association between ΔLAA and mean annual change in peak $\dot{\text{V}}$ co_2_ (Δp$\dot{\text{V}}$ co_2_) is shown in Fig 6. There was a significant correlation between ΔLAA and Δp$\dot{\text{V}}$ co_2_ (p<0.0001, r = -0.488), suggesting that CO_2_ production could decline in proportion to the extent of emphysematous changes.

**Fig 7:**
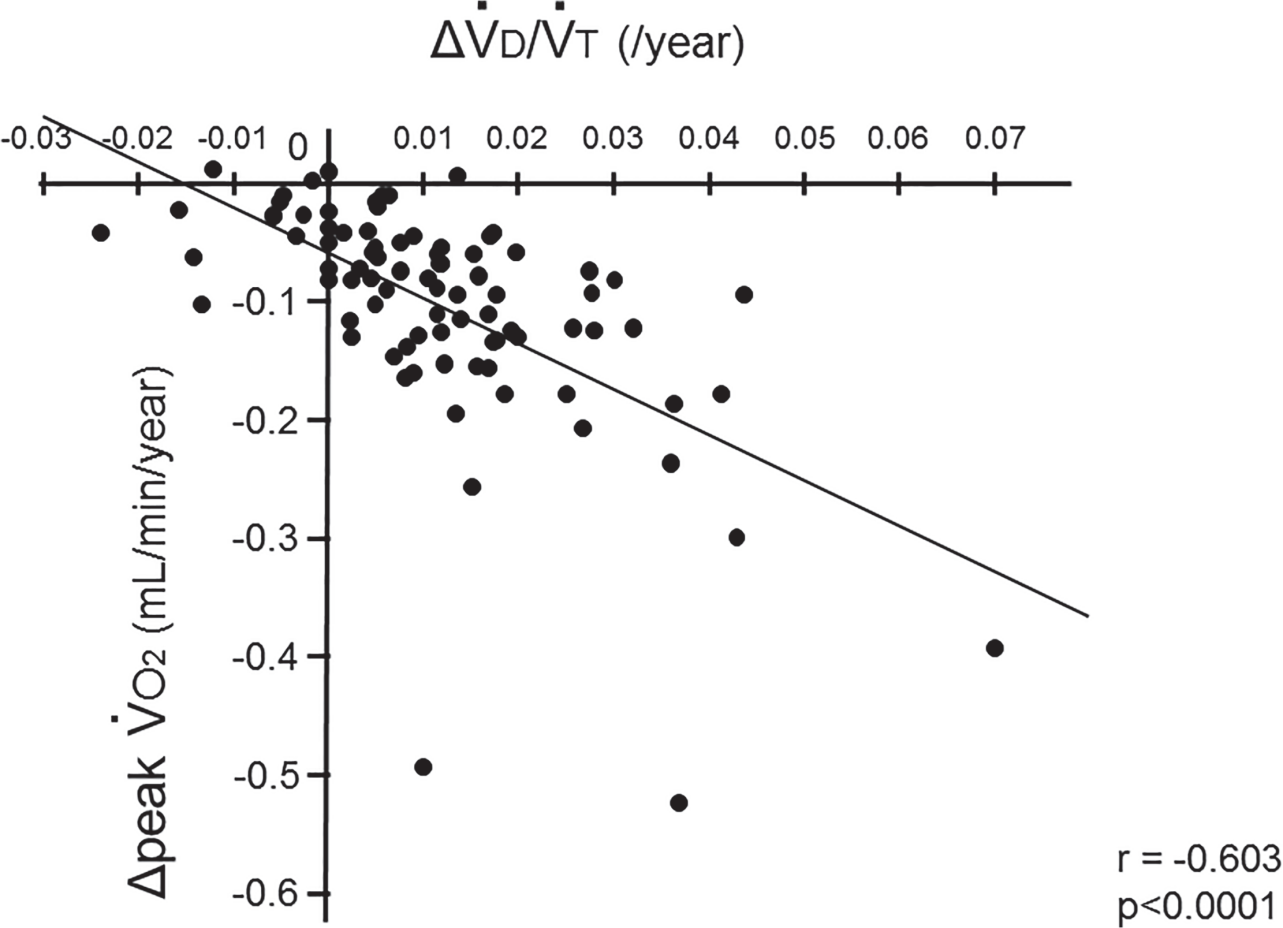
Relationship between the decline in aerobic capacity and the increase in dead space. The mean annual changes in peak $\dot{\text{V}}$ o_2_ (Δp$\dot{\text{V}}$ o_2_) were significantly correlated with those in the dead space fractions (Δ$\dot{\text{V}}$ d/$\dot{\text{V}}$ t) (p<0.0001, r = -0.603).

**Fig 8:**
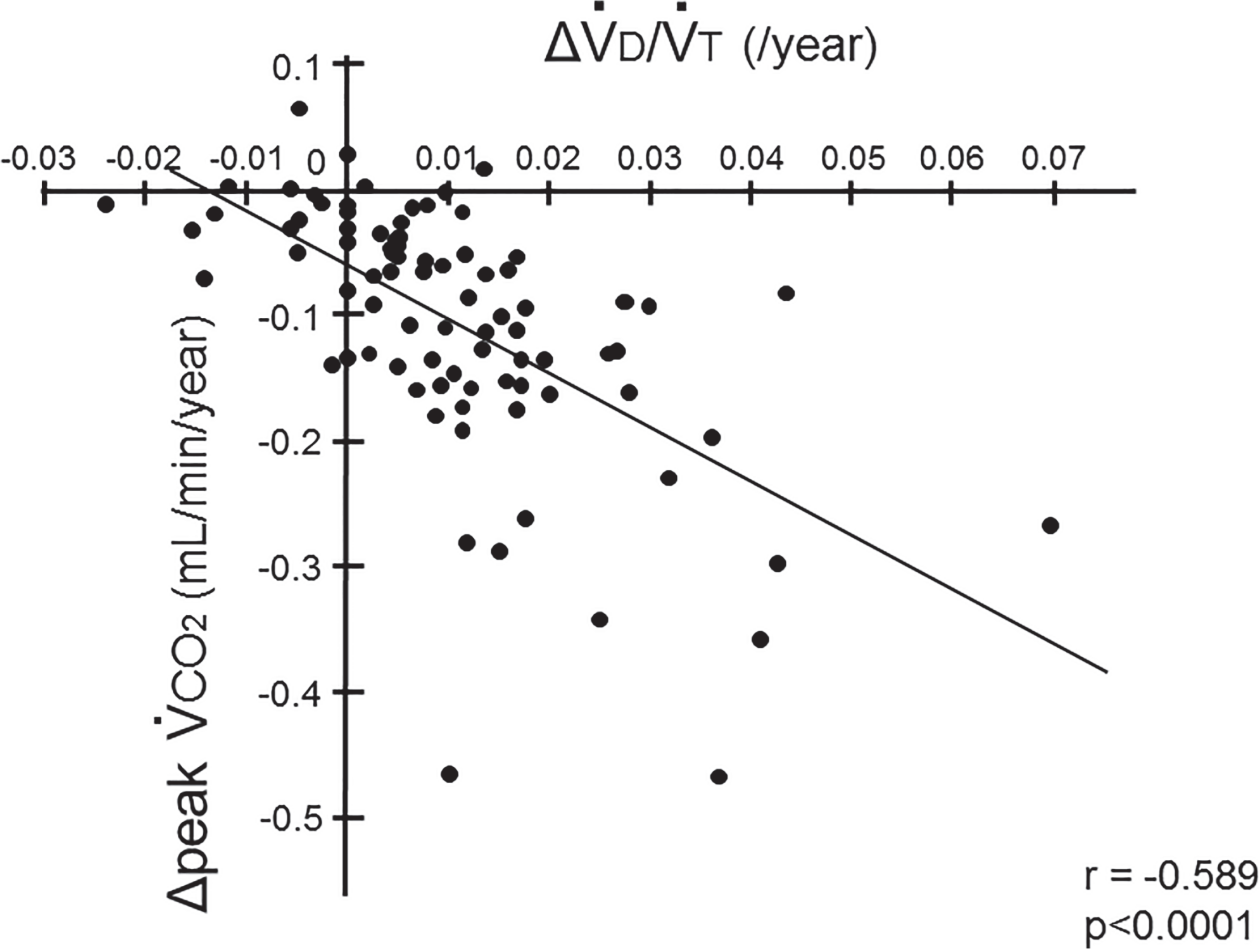
Association between the decline in CO2 production and the increase in dead space. The mean annual changes in peak $\dot{\text{V}}$ co_2_ (Δp$\dot{\text{V}}$ co_2_) were significantly correlated with those in the dead space fractions (Δ$\dot{\text{V}}$ d/$\dot{\text{V}}$ t) (p<0.0001, r = -0.589).

There was no significant correlation between the mean annual change in $\dot{\text{V}}$ d/$\dot{\text{V}}$ t (Δ$\dot{\text{V}}$ d/$\dot{\text{V}}$ t) and ΔLAA (data not shown). As shown in Fig 7, Δ$\dot{\text{V}}$ d/$\dot{\text{V}}$ t was significantly correlated with Δp$\dot{\text{V}}$ o_2_ (p<0.0001, r = -0.603). In addition, Δ$\dot{\text{V}}$ d/$\dot{\text{V}}$ t was inversely associated with xp$\dot{\text{V}}$ co_2_ (p<0.0001, r = -0.589) (Fig 8). These findings indicate that the increase in dead space might be linked to impairment of aerobic capacity and CO_2_ production in patients with COPD.

The time-dependent change in peak SpO_2_ was not correlated with ΔLAA (data not shown).

### Association between longitudinal changes in lung function parameters and that in LAA score

We examined whether the longitudinal changes in lung function data were associated with that in total LAA score. None of the annual changes in lung function parameters (VC, %VC, FEV_1_, FEV_1_/FVC, %FEV_1_, RV/TLC, DLco, and DLco/Va) was correlated with that in emphysema score on HRCT (data not shown).

### Association between temporal changes in CPET parameters and those in lung function parameters

We also evaluated whether the time-dependent changes in CPET results were associated with those in lung function parameters. There was no significant correlation between the changes in CPET parameters, including aerobic capacity and CO_2_ production, and those in lung function parameters (data not shown).

## Discussion

In the present study, we conducted CPET and HRCT in patients with various grades of COPD having a significant smoking history. It was observed that CPET parameters such as aerobic capacity and CO_2_ production were associated with emphysematous change evaluated by HRCT as well as airflow limitation. In addition, repeated assessment revealed that the longitudinal changes in CPET data were correlated with that in total LAA score on HRCT images. These results indicate that HRCT measures of emphysema might be a good tool for predicting aerobic capacity in patients with COPD. To the best of our knowledge, this is the first study concerning the time-dependent changes in aerobic capacity and CO_2_ production in patients with COPD.

Although HRCT is performed to quantitatively assess both emphysema and airway disease in COPD, the issue of whether HRCT measures of emphysema are associated with exercise capacity is highly controversial. Diaz and colleagues reported that the percentage of low attenuation areas less than a threshold of -950 Hounsfield units (%LAA-950) was inversely associated with 6-minute walk distance (6MWD), suggesting that emphysema had a deleterious impact on exercise capacity [17]. A correlation between CT emphysema and walk distance was also revealed in other earlier studies [7, 15]. In contrast to these studies, Mair and coworkers found no correlation between %LAA-950 and 6MWD [22]. Although 6MWT is easily performed and inexpensive, it provides little insight into the mechanisms of exercise limitation. In addition, 6MWD can be affected by a variety of factors unrelated to cardiopulmonary status and gives only a rough estimate of the general functional status of patients [23]. For more specific assessment of the mechanisms of exercise intolerance, CPET with continuous breath-by-breath gas exchange analysis is favorable. In this study, therefore, we performed CPET and analyzed aerobic capacity and CO_2_ production. The study results confirmed that the extent of emphysema on HRCT images was associated with impairment of aerobic capacity and CO_2_ production in patients with COPD.

In the present study, the longitudinal changes in CPET parameters were shown to be correlated with that in total LAA score on HRCT images, suggesting that the progression of emphysematous changes might elicit a decline in aerobic capacity and CO_2_ production. Whereas many cross-sectional studies assessed emphysematous changes using HRCT [5–7, 10–13], there have been few longitudinal studies evaluating the development of emphysema using HRCT [24].

It was previously reported that a decline in 6MWD was associated with changes in airflow obstruction [25] and mortality in patients with severe COPD [15]. In contrast, we found no correlation between the time-dependent changes in CPET parameters and those in lung function. Since exercise capacity is defined not only by aerobic capacity but also by weakness of respiratory and lower extremity muscles [26], it might be influenced by the systemic effect of COPD [27]. Another possible explanation is that the observation period was not long enough to detect a significant decline in lung function.

There were some subjects in whom aerobic capacity rapidly declined. We defined a rapid decliner as a subject with Δp$\dot{\text{V}}$ o_2_ greater than 1 SD from the mean (i.e. 0.095 L/min/year), allowing us to identify 8 subjects as rapid decliners. We evaluated the characteristics of these patients; however, there were no differences in smoking status, medication, and initial CT scores between the rapid decliners and the other study subjects. In addition, only one of the rapid decliners experienced exacerbation, suggesting that exacerbation of COPD might have little impact on rapid decline in aerobic capacity.

In previous reports, frequent exacerbations of COPD were shown to be associated with a faster annual decline in lung function and exercise capacity [28, 29]. In contrast, Suzuki and colleagues revealed that exacerbation events did not significantly influence the annual decline in FEV_1_ [30]. They observed a much lower exacerbation frequency than in other studies, which is comparable with our findings. Since exacerbation occurred only in a small number of patients, we could not estimate the impact of exacerbations on the time-dependent changes in CPET parameters, LAA score, and lung function. The discrepancy in the frequency of exacerbations between Japan and other geographical regions is expected to be owing to differences in health care systems and socioeconomic status [30]. In addition, a smaller number of patients with severe COPD manifestations defined as GOLD grade III or IV might also be responsible for the low frequency of exacerbations in our observation.

In this study, the time-dependent changes in lung function parameters including diffusing capacity were not correlated with that in total LAA score. In contrast, a previous cross-sectional study showed that quantitative CT measurement of emphysema was associated with spirometric results showing impairment in smokers [31]. In our previous 5-year follow-up study, the results of most pulmonary function tests did not change annually, whereas many of the inspiratory HRCT parameters did [32]. This discrepancy might be due to a time lag between the development of emphysema and the decline in ventilatory parameters and diffusing capacity. Moreover, although we repeated the examination after a median interval of 3.5 years, the observation period might not have been long enough to detect a significant decline in lung function.

The mean annual change in dead space fraction (Δ$\dot{\text{V}}$ d/$\dot{\text{V}}$ t) was not associated with ΔLAA, whereas Δ$\dot{\text{V}}$ d/$\dot{\text{V}}$ t was significantly correlated with Δp$\dot{\text{V}}$ o_2_ and Δp$\dot{\text{V}}$ co_2_. In other words, augmentation of dead space, which was responsible for impairment of aerobic capacity and CO_2_ production, was not fully associated with the development of emphysema measured by HRCT. It is well known that COPD with apparent emphysematous changes is characterized by ventilation-perfusion ($\dot{\text{V}}$ a/$\dot{\text{Q}}$) inequality, forming considerable regions of high $\dot{\text{V}}$ a/$\dot{\text{Q}}$ ratios [33]. Besides emphysematous change, destruction and/or remodeling of the pulmonary vascular bed, which could hardly be detected by HRCT, might result in the development of physiological dead space with high $\dot{\text{V}}$ a/$\dot{\text{Q}}$ ratios, leading to impairment of aerobic capacity and CO_2_ production.

There are several limitations of this study. Firstly, we evaluated the extent of emphysema by visual assessment instead of using %LAA-950. However, since %LAA-950 and visual scores of emphysema by experienced investigators have been shown to correlate well [34], the lack of densitometry was not likely to affect the results of this study.

Secondly, because 6MWT was not conducted, we could not compare the usefulness of CPET and 6MWT directly. Further investigation is absolutely required to address this issue.

Thirdly, this study included only one female patient, although it was not intentional. Although it is not easy to identify the cause why it happened, we consider the following four reasons. 1) A previous epidemiological study in Japan showed that COPD was more prevalent in males than in females [35]. 2) A significant part of the female COPD patients in Japan was demonstrated to have no or limited history of smoking. According to a survey by Japan Tobacco Inc. in 2013, the smoking prevalence was higher in males than in females (32.2% vs 10.5%) in Japan. 3) Since the participants were selected from the subjects referred to the university hospital, there is a possibility that they were apart from the male-female frequency of COPD in general population. 4) A significant part of the female candidate of the study were reluctant to undergo CPET. These might be associated with the dominance of males in our study subjects. It remains to be determined whether the results obtained in the present study can be applied to female patients with COPD.

Fourthly, fat-free mass (FFM), which might be associated with the exercise capacity, was not examined in this study. FFM was shown to remain relatively constant with aging, requiring no age adjustment in the reference value [36]. In addition to structural changes, however, there are changes in intrinsic function of the muscles with age. Overall muscle function in the body decreases by 2% annually [37], which suggests that the decline in the exercise capacity we observed might be partly owing to the decreased muscle strength.

In this study, we did not examine serum α_1_-antitrypsin levels, because α _1_-antitrypsin deficiency is extremely rare in Japanese population [38]. In addition, smoking subjects with α _1_-antitrypsin deficiency develop COPD by middle age [39]. Because our study subjects were old and had a lifelong history of smoking, we think that they were not likely to have α _1_-antitrypsin deficiency.

In conclusion, CPET parameters such as aerobic capacity and CO_2_ production were associated with emphysematous change evaluated by HRCT. In addition, the time-dependent changes in CPET data were correlated with that in total LAA score. These findings indicate that HRCT measures of emphysema might be a good tool for predicting aerobic capacity of patients with COPD.
